# The effect and underlying mechanisms of titanium dioxide nanoparticles on glucose homeostasis: A literature review

**DOI:** 10.1002/jat.4318

**Published:** 2022-03-18

**Authors:** Vida Mohammadparast, Beth L. Mallard

**Affiliations:** ^1^ School of Health Sciences Massey University Wellington New Zealand

**Keywords:** blood glucose homeostasis, insulin resistance, titanium dioxide nanoparticles

## Abstract

Titanium dioxide (TiO_2_) is used extensively as a white pigment in the food industry, personal care, and a variety of products of everyday use. Although TiO_2_ has been categorized as a bioinert material, recent evidence has demonstrated different toxicity profiles of TiO_2_ nanoparticles (NPs) and a potential health risk to humans. Studies indicated that titanium dioxide enters the systemic circulation and accumulates in the lungs, liver, kidneys, spleen, heart, and central nervous system and may cause oxidative stress and tissue damage in these vital organs. Recently, some studies have raised concerns about the possible detrimental effects of TiO_2_ NPs on glucose homeostasis. However, the findings should be interpreted with caution due to the methodological issues. This article aims to evaluate current evidence regarding the effects of TiO_2_ NPs on glucose homeostasis, including possible underlying mechanisms. Furthermore, the limitations of current studies are discussed, which may provide a comprehensive understanding and new perspectives for future studies in this field.

## Abstract 2

Titanium dioxide (TiO_2_) is used as a white pigment in a wide array of products and is generally considered to be toxicologically inert. However, once human beings are exposed to TiO_2_ NPs, they can be absorbed, and accumulated in several vital organs, potentially leading to adverse effects on human health. Recently, the result of some studies showed the possible effect of TiO_2_ NPs on glucose homeostasis. In this review, we evaluate and discuss current evidence regarding the effects of TiO_2_ NPs on glucose homeostasis.

## 1 INTRODUCTION

Titanium dioxide (TiO_2_) is one of the most consumed pigments worldwide (Delgado‐Buenrostro et al., 2015; Giovanni et al., 2015). Various particle‐sized TiO_2_ fractions, including fine (approximately 0.1–2.5 μm) and nanosized (<0.1 μm, primary particles) are produced and used (Dankovic et al., 2007). The estimated global consumption of TiO_2_ was 6.1 million metric tons in 2016 and is forecasted to reach 8.83 million metric tons by 2025 (Loosli et al., 2019). TiO_2_ nanoparticles (NPs) are extensively used in nanomedicine, paints, paper, plastics, water cleanup technology, dermal products, self‐cleaning materials, toothpaste, and as an additive in the food industry (Hext et al., 2005). Total daily intakes of TiO_2_ NPs may vary, depending on age group and location (Weir et al., 2012). Because sweets contain a lot of TiO_2_ NPs compared with other food products, the highest levels of exposure occur in children (Weir et al., 2012). In the United States, the daily intake of TiO_2_ NPs is approximately 0.2–0.7 mg/kg BW in adults, and 1–2 mg/kg BW in children, compared with 1 mg/kg BW in adults and 0.72 to 2–3 mg/kg BW in children respectively in the United Kingdom, suggesting children's intake is up to three times that of adults (Weir et al., 2012). TiO_2_ NPs can enter the body via inhalation (respiratory tract), ingestion (gastrointestinal tract), dermal penetration (skin), and injection (blood circulation) (Oberdörster et al., 2005; Shi et al., 2013). A growing body of evidence has shown that inhaled and ingested TiO_2_ crystals enter the bloodstream (Pele et al., 2015; Zhao et al., 2018).

TiO_2_ NPs were previously categorized as a bioinert material that has minimal detrimental effects on humans and animals (Lindenschmidt et al., 1990; Ophus et al., 1979). However, according to the latest update from the European Food Safety Authority (EFSA) in May 2021, TiO_2_ can no longer be considered as a safe food additive and its genotoxic effects cannot be excluded (EFSA Panel on Food Additives and Flavourings [FAF] et al., 2021). Recent evidence has suggested that TiO_2_ NPs may have different toxicity profiles than fine‐sized TiO_2_ particles due to their different chemical, optical, magnetic, and structural properties (Maynard et al., 2006; Wu et al., 2009). For instance, in most cells exposed to TiO_2_ NPs, a series of morphological changes, including decreased cell size, membrane blebbing, peripheral chromatin condensation, and apoptotic body formation was detected (Gurevitch et al., 2012; Hussain et al., 2010). Some studies have seen the accumulation of absorbed TiO_2_ NPs in the lungs, liver, kidneys, spleen, heart, and central nervous system (Grande & Tucci, 2016; Younes et al., 2015). In a study using cadaveric human liver samples, half of the subjects had TiO_2_ NPs accumulation above the level considered safe in the liver (Heringa et al., 2018). The small size and difficult clearance of TiO_2_ NPs make them cytotoxic and cause oxidative stress and tissue damage in these vital organs (Alarifi et al., 2013; Grande & Tucci, 2016; Younes et al., 2015). In mouse studies, TiO_2_ particles promote transcription of pro‐inflammatory cytokines, such as TNF‐α, IL‐6, and IL‐1β (Cui et al., 2011; Park et al., 2009; Trouiller et al., 2009). Intriguingly, pro‐inflammatory cytokines are a well‐known factor in inducing insulin resistance and linking to the pathogenesis of type 2 diabetes (T2D) and obesity (Nov et al., 2010; Olefsky & Glass, 2010; Shoelson et al., 2006). Conversely, in rats, acute and chronic dietary consumption of E 171 neither induced histologic changes in liver, spleen, small and large intestines, and lungs nor triggered blood inflammatory cytokine production (Blevins et al., 2019).

The expected values for normal plasma glucose levels are less than 100 mg/dl during fasting and under 140 mg/dl 2‐h postprandial (Gurung & Jialal, 2021). Under normal circumstances, physiological control mechanisms tightly match the uptake of glucose by tissues and the appearance of glucose in the bloodstream (Wolfe & Chinkes, 2004). The maintenance of blood glucose homeostasis is always of primary importance and is required for the optimum function of the brain and nervous system (Suh et al., 2007). There is some evidence indicating that NPs can disrupt glucose metabolism. Shin et al. (2019) reported that NPs increased reactive oxygen species (ROS) and caused reduced glucose uptake and alteration in the glucose metabolic function. Engineered nanomaterials (ENMs) may decrease insulin sensitivity and damage pancreatic beta (β)‐cells, thus changing glucose homeostasis, which might lead to type 2 diabetes mellitus (T2DM) (Priyam et al., 2018). Findings from a nested case–control study indicated a robust positive association between titanium intake and incident diabetes risk (Yuan et al., 2018). The association of urinary titanium with the risk of impaired fasting glucose (IFG) and fasting plasma glucose (FPG) levels was reported in a cross‐sectional survey (Feng et al., 2015). Hu et al. (2018) in their in vivo tests on mice demonstrated that oral administration of TiO_2_ NPs induces the imbalance of glucose homeostasis. Similar results were obtained by Mao et al. (2019), in which prenatal exposure to TiO_2_ NPs led to an increase in maternal fasting blood glucose levels. In a study by Chen et al. (2018), orally administrated TiO_2_ NPs caused a slight and temporary hypoglycemic effect in rats. Recently, Heller et al. (2018) have detected TiO_2_ crystals in pancreatic specimens from type 2 diabetics; in contrast, crystals were not found in the nondiabetic group, raising the possibility of the association of TiO_2_ exposure and the pathogenesis of T2D. In light of these studies, we reviewed the current literature that has specifically investigated the effects of TiO_2_ NPs on glucose homeostasis and the possible mechanisms for its influence.

## 2 METHODS

### 2.1 Data source and search strategy

Several databases, including Science Direct, PubMed, and Google Scholar were searched to identify any related articles until August 2021. The Medical Subject Heading (MeSH) terms: (((“diabetes mellitus”[MeSH Terms] OR (“diabetes”[All Fields] AND “mellitus”[All Fields]) OR “diabetes mellitus”[All Fields]) AND ((“glucose”[MeSH Terms] OR “glucose”[All Fields] OR “glucoses”[All Fields] OR “glucose s”[All Fields]) AND (“homoeostasis”[All Fields] OR “homeostasis”[MeSH Terms] OR “homeostasis”[All Fields]))) OR (“insulin resistance”[MeSH Terms] OR (“insulin”[All Fields] AND “resistance”[All Fields]) OR “insulin resistance”[All Fields]) OR (“insulin resistance”[MeSH Terms] OR (“insulin”[All Fields] AND “resistance”[All Fields]) OR “insulin resistance”[All Fields] OR (“insulin”[All Fields] AND “sensitivity”[All Fields]) OR “insulin sensitivity”[All Fields])) AND ((“titanium dioxide”[Supplementary Concept] OR “titanium dioxide”[All Fields]) AND (“nanoparticle s”[All Fields] OR “nanoparticles”[MeSH Terms] OR “nanoparticles”[All Fields] OR “nanoparticle”[All Fields])) were used for collecting data from PubMed, while for other databases combinations of the following keywords had been applied including “Diabetes Mellitus,” “Glucose Homeostasis,” “Insulin Resistance,” “Insulin Sensitivity,” and “Titanium dioxide nanoparticles.”

### 2.2 Inclusion criteria and exclusion criteria

Original studies that examine the effect of TiO_2_ NPs on glucose homeostasis and explained the possible mechanisms were selected. The chosen articles were limited to articles published in the English language. Moreover, we did not limit the type of studies included, and all sorts of original studies (in vivo, in vitro, human study) were considered. Articles were excluded if they were editorials, poster abstracts, or had no full text available.

### 2.3 Data management

**FIGURE 1:**
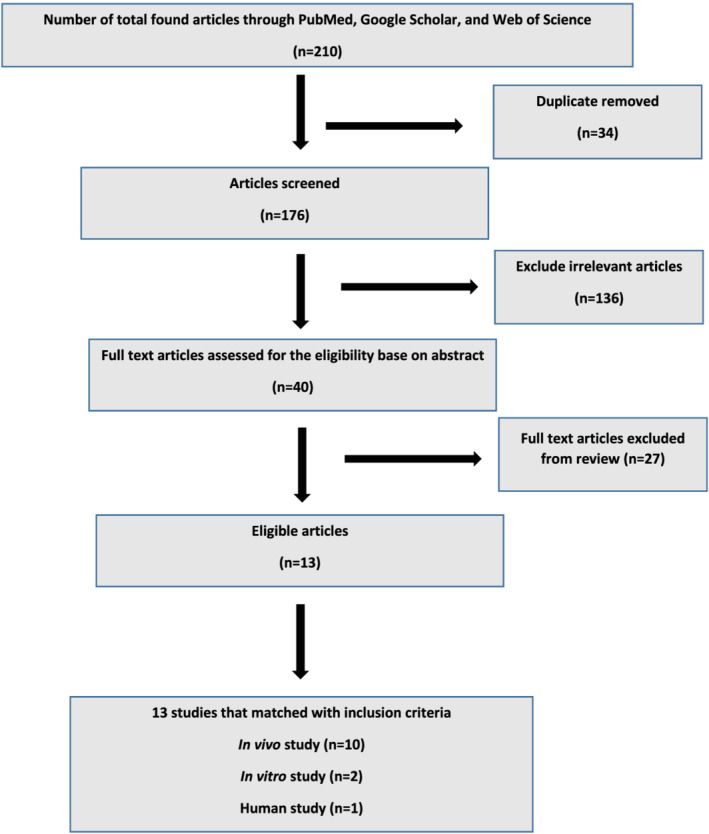
Flowchart of study selection process [Colour figure can be viewed at wileyonlinelibrary.com]

Our initial search started with a screening of the 210 papers. After removing duplicates and irrelevant studies according to their titles and abstracts, the eligibility of the remaining 40 papers was reviewed based upon their full text. Ultimately, 13 studies were included in this review by assessing their full texts (Figure 1).

Table 1 outlines information of eligible articles, including animal model, TiO_2_ NPs doses, exposure time, result, and suggested mechanisms for the influence of TiO_2_ NPs on glucose homeostasis.

**TABLE 1:** A short review of the conducted studies

Model	Type of TiO_2_ NPs	Dose (mg/kg)	Route of exposure	Exposure time (weeks)	Result	Ref.
CD‐1 (ICR) mice	Anatase TiO_2_	0.52, 2.6, 13, 64, and 320	Oral administration via a syringe	14	‐ Disturbance of glucose homeostasis ‐ Activation of inflammatory pathways including NF‐κB, JNK, and MAPK pathways ‐ Increased levels of IL‐6 and TNF‐α ‐ Increase in the reactive oxygen species production	(37) (Hu et al., 2015)
CD‐1 (ICR) mice	Anatase/rutile TiO_2_	64	Oral administration via a syringe	18	‐ Disturbance of glucose homeostasis ‐ Increase in the reactive oxygen species production	(38) (Hu et al., 2016)
CD‐1 (ICR) mice	Anatase/rutile TiO_2_	10, 20, 50, 100, and 200	Oral administration via a syringe	26	‐ An impairment of insulin signaling pathway ‐ Activation of inflammatory pathways including NF‐κB, JNK, and MAPK pathways ‐ Increased serum levels of IL‐6 and TNF‐α ‐ Induction of cytochrome P‐450 gene expression ‐ Increase in expression of ER stress‐related genes and protein ‐ Induction of the expression of genes related to the unfolded protein response (UPR)	(39) (Hu et al., 2018)
CD‐1 (ICR) mice	Unknown	50	Oral administration via a syringe	26	‐ Disturbance of glucose homeostasis ‐ Increased ROS level ‐ Induction of cytochrome P‐450 gene expression ‐ Increase in expression of ER stress‐related genes and protein ‐ Induction of the expression of genes related to the UPR	(40) (Hu et al., 2020)
CD‐1 (ICR) mice	Anatase TiO_2_	64	Oral administration via a syringe	28	‐ Disturbance of glucose homeostasis ‐ An impairment of insulin signaling pathway ‐ Increased levels of IL‐6 and TNF‐α ‐ Increase in the reactive oxygen species production	(41) (Gu et al., 2015)
SD rats	Anatase TiO_2_	0, 2, 10, and 50	Oral gavage	30 or 90 consecutive days	‐ A slight and temporary hypoglycemic effect ‐ Reduced length and number of small intestinal villi ‐ Reducing the area available for absorption in the small intestine	(42) (Chen et al., 2018)
Fao rat hepatoma cells, murine macrophage cell line (J774.1)	Anatase TiO_2_	100	‐	2 h	‐ Inducing insulin resistance ‐ Up‐regulation of pro‐inflammatory genes expression including TNF‐α, IL‐1α, IL‐1β, IL‐6, and IL‐8 ‐ Activation of stress kinases, MAPK38, and JNKs	(15) (Gurevitch et al., 2012)
CD‐1 (ICR) mice, NIH/3T3 cells	Anatase/rutile TiO_2_	50 and 0.1	Oral administration via a syringe	26	‐ Disturbance of glucose homeostasis ‐ An impairment of insulin signaling pathway ‐ Increased ROS production	(43) (Hu et al., 2019)
SD rats	Anatase TiO_2_	0, 2, 10, and 50	Oral gavage	90 consecutive days	‐ Mild and temporary hypoglycemia ‐ Decreased blood insulin and C‐peptide	(44) (Chen et al., 2020)
SD rats	Unknown	5 mg/kg	Oral gavage	From the 5th to 18th day after pregnancy	‐ Raised fasting blood glucose ‐ Alterations of gut microbiota ‐ The enhancement of the type 2 diabetes mellitus related genes ‐ Reduction of taurine and hypotaurine metabolism	(45) (Mao et al., 2019)
Human epithelial colorectal adenocarcinoma (Caco‐2) cell line, human intestinal goblet cell (HT29‐MTX) line, *Drosophila melanogaster*	Anatase TiO_2_	1.4 × 10^−4^ mg/ml, 5, 50, and 500 ppm	‐	4 h, from first instar larvae to adulthood	‐ Decrease plasma glucose concentrations ‐ Transformation in microvilli ‐ Decreased transport of glucose across the gut epithelium	(46) (Richter et al., 2018)
SD rats	TiO_2_ NPs	2, 10, or 50	Intragastric administration	30 days	‐ Damage to the intestinal villi and microvilli structures ‐ No change in blood glucose level	(47) (Gao et al., 2020)

Abbreviations: ER stress, endoplasmic reticulum stress; IL‐1β, interleukin 1 beta; IL‐6, interleukin 6; IL‐8, interleukin 8; JNK, c‐Jun N‐terminal kinase; MAPK, mitogen‐activated protein kinase; NF‐κB, nuclear factor kappa B; TNF‐α, tumor necrosis factor‐α.

**FIGURE 2:**
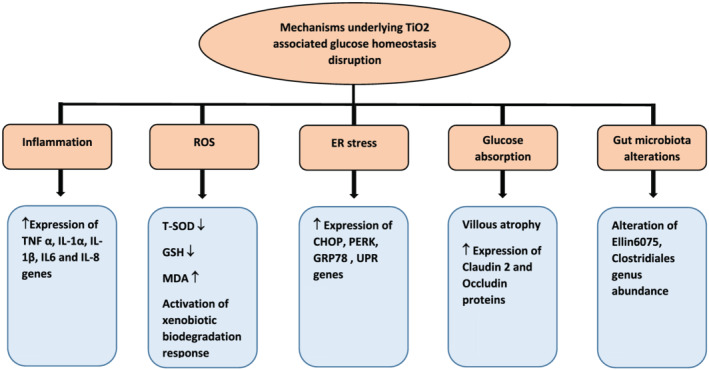
Summary of the reported potential mechanisms underlying the association of TiO_2_ NPs and glucose homeostasis [Colour figure can be viewed at wileyonlinelibrary.com]

The mechanisms by which TiO_2_ was reported to influence glucose homeostasis are summarized in Figure 2.

## 3 FINDINGS

TiO_2_ NPs exposure impaired glucose homeostasis and induced insulin resistance, either directly by influencing hepatic glucose metabolism and intestinal glucose absorption, interfering with insulin signaling pathway (Gurevitch et al., 2012; Richter et al., 2018), and/or indirectly by increasing ROS, endoplasmic reticulum (ER) stress, and activation inflammatory pathways (Hu et al., 2015, 2016, 2018, 2020). The association of TiO_2_ NPs and disrupted glucose homeostasis and underlying mechanisms are explained in greater detail in the following paragraphs.

Oral administration of TiO_2_ NPs, at doses of at least 10 mg/kg, to mice results in the accumulation of Ti even in several organs, including the liver and pancreas (Gu et al., 2015; Gurevitch et al., 2012; Hu et al., 2015, 2016, 2018, 2020). In rats, however, there is no significant accumulation of Ti at doses of 50 mg/kg (Chen et al., 2018; Chen, Zhou, Zhou, et al., 2019). Despite an absence of accumulation of Ti in rat liver, TiO_2_ NPs induce fatty degeneration of hepatocytes, although this hepatocellular damage is not reflected by serum markers of liver injury and function (Chen et al., 2020; Chen, Zhou, Han, et al., 2019; Chen, Zhou, Zhou, et al., 2019).

Gurevitch et al. (2012) evaluated the potential toxic effect of TiO_2_ NPs on insulin resistance in liver‐derived cells, Fao cells (rat hepatoma), and found that TiO_2_ NPs stimulated insulin resistance, indicated by impaired glycogen synthesis and reduced activation of the insulin signaling pathway. Similarly, in mice, TiO_2_ NPs disturbed plasma glucose homeostasis, marked by increased plasma glucose levels, and impaired glucose tolerance, with no difference in plasma insulin levels compared with untreated controls (Gu et al., 2015; Hu et al., 2015, 2016, 2018, 2019, 2020), TiO_2_ impaired activation of components of the insulin signaling pathway in mice (Gu et al., 2015; Hu et al., 2018, 2019, 2020). Additionally, in mice that were immature at the onset of TiO_2_ NP administration, changes in plasma glucose levels and reduced glucose tolerance were induced by 8 weeks of treatment, whereas in mice commencing TiO_2_ treatment as adults, this disturbance was delayed until 26 weeks (Hu et al., 2020). Conversely, in rats, no significant change in plasma glucose levels or oral glucose tolerance test (OGTT) results was observed following treatment with TiO_2_ NPs (Chen et al., 2018). Furthermore, a 90‐day oral toxicity study of concurrent treatment with TiO_2_ NPs and glucose in rats showed a significant decrease in plasma glucose area under the curve (AUC), the level of HbA1c, insulin, and an increase in the level of glucagon in female rats compared with controls, but male rats treated with the same regimen displayed elevated plasma glucose at 60 min and increased plasma glucose AUC in the OGTT and decreased in the level of plasma insulin and C‐peptide, and glucagon (Chen et al., 2020). These results indicate that there are age, species, and sex differences in the effects of TiO_2_ NPs.

Pro‐inflammatory cytokines play a role in insulin resistance. In a macrophage cell line (J774.1), TiO_2_ up‐regulated the expression of genes for pro‐inflammatory cytokines including TNF‐α, IL‐1α, IL‐1β, IL‐6, and IL‐8 (Gurevitch et al., 2012). Furthermore, direct interaction of the TiO_2_ NPs with the liver‐derived cells leads to activation of the stress kinases, mitogen‐activated protein (MAP) kinases p38 (MAPK38) and c‐Jun N‐terminal kinases (JNKs), resulting in the induction of abnormal insulin signaling (Gurevitch et al., 2012). In mice, oral administration of TiO_2_ NPs leads to activation of inflammatory pathways including NF‐κB, JNK, and MAPK pathways (Hu et al., 2015, 2018, 2020) and increased serum levels of IL‐6 and TNF‐α (Gu et al., 2015; Hu et al., 2015, 2016). Mice beginning chronic TiO_2_ NPs exposure as adults took longer to show evidence of inflammation than mice receiving TiO_2_ prior to maturity (Hu et al., 2020).

ROS are believed to play a role in the pathogenesis of insulin resistance (Houstis et al., 2006). Treatment with TiO_2_ NPs has been shown to increase ROS production. This was shown by reduced in the liver and serum levels of total superoxide dismutase (T‐SOD) and glutathione synthetase (GSH) and increased methane dicarboxylic aldehyde (MDA) which together indicates increased ROS (Gu et al., 2015; Hu et al., 2015, 2016, 2019, 2020). RNA‐seq and RT‐qPCR showed that titanium dioxide increases gene expression of components of the cytochrome P450 superfamily, which is involved in ROS production. Two studies using RNA‐seq have identified that in livers from mice chronically exposed to TiO_2_ NPs, a xenobiotic biodegradation response is activated (Hu et al., 2019, 2020), including increased expression of genes related to the cytochrome P450 family. This increased expression was confirmed by RT‐qPCR (Hu et al., 2018, 2019, 2020) and was shown to occur before changes in plasma glucose and glucose tolerance were evident, verifying that high plasma glucose did not stimulate the increase in ROS (Hu et al., 2020). Resveratrol, which is a polyphenol and a ROS scavenger, has been shown in mice to prevent TiO_2_ NP‐induced changes in glucose tolerance plasma glucose levels and also suppresses titanium dioxide induced changes in expression of genes involved in ROS generation pathways (Hu et al., 2019). Taken together, this suggests that TiO_2_ NPs induce ROS production, at least in part via a xenobiotic biodegradation response, which then contributes to the induction of insulin resistance.

TiO_2_ NPs administration has been shown to increase the expression of genes and proteins related to ER stress, such as CHOP, PERK, and GRP78, and also induce the expression of genes related to the unfolded protein response (UPR) (Hu et al., 2018, 2020). Relief of ER stress with phenyl butyric acid (4‐PBA) has been shown to prevent TiO_2_ NP‐induced changes in glucose tolerance and insulin signaling (Hu et al., 2018, 2019). 4‐PBA suppressed both the TiO_2_ NP‐induced changes in ER stress markers and maintained ROS at control levels (Hu et al., 2019). Chronic administration of TiO_2_ NPs in juvenile mice induces increased expression of ER stress‐related genes and proteins by 8 weeks of TiO_2_ NPs exposure, whereas in adult mice, the ER stress‐related gene and protein expression changes are delayed but are present after 26 weeks exposure (Hu et al., 2020). Resveratrol treatment prevents the TiO_2_ NP‐induced expression of ER stress and UPR genes (Hu et al., 2019). Therefore, a possible mechanism for the loss of glucose homeostasis and disruption of insulin signaling in mice chronically exposed to TiO_2_ NPs is that the TiO_2_ NPs induce an increase in expression of cytochrome P450 in the liver, leading to ER stress and consequent inflammation leading to insulin resistance and loss of blood glucose control (Hu et al., 2019).

Richter et al. (2018) assessed the effects of exposure to TiO_2_ NPs on the transfer of glucose across the small intestine by using the in vitro culture models of the small intestine (Caco‐2, HT29‐MTX cell line) and *Drosophila melanogaster* as an in vivo model. Acute exposure to TiO_2_ NPs concentrations caused a significant decrease in transport of glucose across the gut epithelium due to transformation in microvilli. These findings were verified by a complementary study in *D. melanogaster* where TiO_2_ ingestion caused reduced glucose content. In rats exposed to TiO_2_ NPs for 30 days, small intestinal structure was altered, with villous atrophy, indicated by reduced length and number of small intestinal villi, reducing the area available for absorption (Chen et al., 2018). In addition to alterations in the small intestinal villi structure, TiO_2_ was also shown to increase the expression of the tight junction proteins Claudin 2 and Occludin (Chen et al., 2018). On the contrary, Gao et al. (2020) have shown that administration of different sizes of TiO_2_ NPs (24 and 120 nm) at varying doses (2, 10, or 50 mg/kg BW) for 30 consecutive days leads to no obvious differences in the blood glucose level despite TiO_2_ NP‐induced intestinal epithelium injury.

In the study by Mao et al., the effect of TiO_2_ NPs during pregnancy was examined. It was observed that the rats' fasting glucose levels raised significantly at gestation day (GD) 10 and GD17 after exposure to TiO_2_ NPs, which they hypothesized might be caused by gut microbiota. They identified the flora composition, and the abundance of specific genera, including Ellin6075, *Clostridiales*, and *Dehalobacteriaceae*, was altered in the second trimester and late pregnancy stage. The output of phylogenetic investigation of communities by reconstruction of unobserved states (PICRUSt) has shown the enhancement of the T2DM‐related genes and reduction of taurine and hypotaurine metabolism at the second trimester in TiO_2_ NP‐treated rats (Mao et al., 2019).

In a pilot study, Heller et al. examined 11 pancreas specimens, eight specimens from the donors suffering from T2D, and three from nondiabetic donors. They found TiO_2_ crystals in all pancreas specimens with T2D, while the crystals were not detected in the nondiabetic donor tissue. Based on the result, they concluded T2D might be a chronic crystal‐associated inflammatory disease of the pancreas (Heller et al., 2018).

## 4 DISCUSSION

To the best of our knowledge, the present review was the first article reviewing the effects of TiO_2_ NPs on glucose homeostasis and the underlying mechanisms. Previous review articles (Iavicoli et al., 2011, 2012; Shi et al., 2013) focused on the toxic effects induced by TiO_2_ NPs on organ systems.

Several studies demonstrated that exposure to TiO_2_ NPs induced insulin resistance and increased plasma glucose levels (Gu et al., 2015; Gurevitch et al., 2012; Hu et al., 2015, 2016, 2018, 2019, 2020). Increased accumulation of ROS, the induction of ER stress, activation of the inflammatory response, and MAPK pathways have been considered the predominant pathogenic mechanisms initiated by TiO_2_ NPs. These mechanisms may be involved in hyperglycemia by phosphorylation of serine/threonine in insulin receptor substrate 1 (IRS1) in liver cells leading to the decline in insulin sensitivity, resulting in insulin resistance (Gu et al., 2015; Gurevitch et al., 2012; Hu et al., 2015, 2016, 2018, 2019, 2020). Other studies reported that TiO_2_ NPs exposure led to hypoglycemia and decreased glucose concentrations through an increase in the level of hepatic glucose metabolism and intestinal villi atrophy. The increased levels of hepatic glucose metabolism promote increased glucose consumption and decrease blood glucose levels. The villus atrophy causes a reduction in the intestinal absorption of glucose, possibly through a decrease in the total amount of glucose transporter proteins and an increase in the expression of tight junction proteins, which decrease passive transport of glucose (Chen et al., 2018, 2020; Richter et al., 2018).

While studies in mice (Hu et al., 2016, 2018, 2020) have shown a significant hyperglycemic effect of TiO_2_ NPs, TiO_2_ exposure in rats had either a slight hypoglycemic effect or no obvious change in the blood glucose levels (Chen et al., 2018, 2020; Gao et al., 2020). It is possible that these conflicting results have been related to methodological issues. First, different experimental subjects may influence the dose–response relationship. A study by Graham et al. (2017) has demonstrated that the relationship between dose and response is complicated by the dynamic chemical and physical transformation in the NPs induced by the biological system, leading to an altered response. Second, TiO_2_ NPs were administered to rats and mice through different routes, oral gavage and oral syringe, respectively. Administration of chemical substances via gavage in comparison with other oral routes leads to different toxicokinetic profiles (Graham et al., 2017). Gavage exposures avoid the oral mucosa interactions that influence its absorption, bioavailability, and metabolism (Graham et al., 2017). For instance, administration of donepezil to rodents via gavage causes a lower concentration of the drug in the blood and brain compared with when it is consumed from the oral syringe (Du et al., 2012). Also, the use of gavage administration can confound the assessment of any chemical by inducing stress responses, thus altering any endocrine‐responsive endpoint (Vandenberg et al., 2014). In addition, delivering TiO_2_ in a once per day bolus, which occurred in both species, does not reflect human TiO_2_ consumption in food.

Furthermore, the only human study in this review has shortcomings that deserve more attention. According to the pilot study (Heller et al., 2018), the high count of TiO_2_ crystals abound in type 2 diabetes with pancreatitis (T2Dp) pancreas raises the possibility that T2D could be crystal‐caused inflammatory pancreas disease. However, it is highly possible that the formation of these crystals is a result of inflammation of the pancreas. Previous studies have shown that chronic pancreatitis gave rise to the development of different crystal types, including calcium carbonate crystals in the form of calcite (Multigner et al., 1985) and calcium oxalate crystals (Cartery et al., 2011). It must also be noted that this pilot study had a small sample size and did not consider potential confounding risk factors like family history, so should be interpreted with caution.

Extrapolation of current research data to humans has limitations that preclude drawing unequivocal conclusions. Most of the studies discussed in this review explored the influence of TiO_2_ NPs on glucose homeostasis, applying unrealistic doses in short‐term exposure that may not be relevant to human exposure to TiO_2_. For evaluation of actual exposure in the population, we need long‐term studies, assessing realistic doses. Also, although there are various exposure routes for TiO_2_ NPs to enter the body such as inhalation, dermal exposure, intravascular injection, and oral ingestion (Shi et al., 2013), only the impact of orally administered TiO_2_ NPs on glucose homeostasis has been investigated to date. The effect on glucose homeostasis of TiO_2_ via different routes of exposure certainly warrants further investigation.

Additionally, despite the increased production and utilization of TiO_2_ NPs in the past decade, epidemiological studies that examine the effect of TiO_2_ NPs on glucose homeostasis have not been reported. Epidemiologic investigations with large‐scale settings will be needed to assess the link between exposure to TiO_2_ NPs and alterations to glucose homeostasis and reach a definite conclusion. The exact molecular mechanisms by which TiO_2_ NPs may interrupt glucose homeostasis are unclear. Based on these limited data, ROS generation, ER stress, and inflammatory response may be the main reason for disturbing glucose homeostasis. In addition, all these in vivo studies have been carried out on small rodents such as rats and mice, which may not be optimum for scrutinizing the toxic effects of the nanomaterial over the long term and makes it challenging to obtain results that be illustrative of human exposure/effects (Priyam et al., 2018).

## 5 CONCLUSION

Considering increasing global manufacturing and the potential applications of TiO_2_ NPs in many fields, it is expected that the humans will be exposed to increased levels of these NPs. Therefore, their effect on human health must be further evaluated. There is some evidence that TiO_2_ NPs may alter glucose regulation; however, this evidence is inconsistent. The information collected from current literature indicates methodological heterogeneity in terms of type of TiO_2_ NPs, methods for nanomaterial characterization, doses used, route of exposure, and measurement techniques. There is also evidence that TiO_2_ may have varied effects depending on the species, gender, and developmental stage of the experimental subject. The current knowledge in this field underscores the need for additional studies to investigate the toxicity of TiO_2_ NPs on glucose regulation. Consequently, we recommend that future studies (1) assess the potentially toxic effects of TiO_2_ NPs using more realistic low‐dose through various routes of exposure; (2) apply a thorough and homogeneous exposure classification to elucidate the association of chemical and physical properties of TiO_2_ NPs with their adverse effect on glucose homeostasis; (3) carry out the epidemiological investigations on exposed consumers and workers to identify the possible correlation of human exposure to TiO_2_ NPs and risk of glucose homeostasis disruption; and (4) clarify the potential molecular mechanisms behind the toxicity of TiO_2_ NPs as the underlying molecular mechanisms are still mostly unknown.

## CONFLICT OF INTEREST

The authors have no conflict of interest to report.

## Data Availability

Data sharing is not applicable to this article as no datasets were generated or analyzed during the current study.
